# Repeat expansion scanning of the *NOTCH2NLC* gene in patients with multiple system atrophy

**DOI:** 10.1002/acn3.51021

**Published:** 2020-04-06

**Authors:** Pu Fang, Yanyan Yu, Sheng Yao, Shuyun Chen, Min Zhu, Yunqing Chen, Keji Zou, Lulu Wang, Huan Wang, Ling Xin, Tao Hong, Daojun Hong

**Affiliations:** ^1^ Department of Neurology The First Affiliated Hospital of Nanchang University Nanchang China; ^2^ Department of Neurology The Sixth Medical Center of PLA General Hospital Beijing China; ^3^ Department of Neurology Affiliated Hospital of Guiyang Medical University Guiyang China; ^4^ Department of Health, Exercise Science, and Recreation Management University of Mississippi University Mississippi USA; ^5^ Department of Neurosurgery The First Affiliated Hospital of Nanchang University Nanchang China

## Abstract

**Objective:**

Trinucleotide GGC repeat expansion in the 5’UTR of the *NOTCH2NLC* gene has been recognized as the pathogenesis of neuronal intranuclear inclusion disease (NIID). Previous studies have described that some NIID patients showed clinical and pathological similarities with multiple system atrophy (MSA). This study aimed to address the possibility that GGC repeat expansion in *NOTCH2NLC* might be associated with some cases diagnosed as MSA.

**Methods:**

A total of 189 patients with probable or possible MSA were recruited to screen for GGC repeat expansion in *NOTCH2NLC* by repeat‐primed PCR (RP‐PCR). In addition, long‐read sequencing (LRS) was performed for all patients with RP‐PCR‐positive expansion, five patients with RP‐PCR‐negative expansion, and five controls on the Nanopore platform. Skin biopsies were performed on two patients with GGC expansion.

**Results:**

Five of 189 patients (2.6%) were found to have GGC expansion in *NOTCH2NLC*. LRS results identified that the five patients had GGC expansion between 101 and 266, but five patients with RP‐PCR‐negative expansion and five controls had GGC expansion between 8 and 29. Besides the typical symptoms and signs of MSA, patients with GGC expansion might have longer disease duration, severe urinary retention, and prominent cognitive impairment. In the skin samples from the patients with GGC expansion, typical p62‐postive but alpha‐synuclein‐negative intranuclear inclusions were found in fibroblasts, adipocyte and ductal epithelial cells of sweat glands.

**Conclusion:**

Trinucleotide GGC repeat expansion in *NOTCH2NLC* could be observed in patients with clinically diagnosed MSA. Adult‐onset NIID should be considered as a differential diagnosis of MSA.

## Introduction

Recently trinucleotide GGC repeat expansion in the 5′‐untranslated region (5′UTR) of the *NOTCH2NLC* gene has been recognized to be involved in the pathogenesis of neuronal intranuclear inclusion disease (NIID).^1^, ^2^, ^3^, ^4^ The neurological manifestations of adult‐onset NIID have a high degree of clinical heterogeneity, and usually include autonomic dysfunctions, parkinsonism, cerebellar ataxia, cognitive impairments, encephalitic episode, pyramidal signs, and sensory disturbances.^5^ Neuropathological examination of NIID patients has demonstrated the widespread presence of eosinophilic intranuclear inclusions in the cells of central and peripheral nervous systems as well as other organs.^6^

Multiple system atrophy (MSA) is a deteriorating neurodegenerative disorder pathologically characterized by alpha‐synuclein‐positive glial cytoplasmic inclusions in multiple brain areas.^7^ Clinical features of MSA mainly comprise dysautonomia, parkinsonism, cerebellar ataxia, and pyramidal signs in various combinations.^8^ Regarding clinical presentation, MSA appears to be autonomic failure with parkinsonism (MSA‐P), cerebellar ataxia (MSA‐C), or both.^9^ Previous studies have described that some NIID patients showed clinical and pathological similarities with those of MSA.^10^, ^11^ Therefore, adult‐onset NIID appears to be a possible phenocopy of MSA and thus a potentially important differential diagnosis of MSA. There is considerable uncertainty about the significance of this clinical overlap, especially given the possible genetic implications of NIID and the poor prognosis of MSA compared with NIID.^12^, ^13^ To address these questions, we screened the *NOTCH2NLC* gene in 189 sporadic patients with clinically diagnosed MSA and 325 healthy adults with the aim of identifying the possible association between MSA and GGC repeat expansion of the *NOTCH2NLC* gene.

## Materials and Methods

### Subjects

One hundred eighty‐nine patients with sporadic MSA visiting in any of the three academic neurological centers between July 2015 and July 2019 were retrospectively recruited for this study. The inclusion criteria included: (1) probable or possible MSA;^9^ (2) no family history of MSA and other neurological disorders; and (3) DNA samples available. MSA patients were identified from searching the electronic medical record using International Classification of Diseases, tenth Revision (ICD10) code G90.302. Clinical information, including gender, age, age of onset, progression of disease, family history, and other clinical manifestations, was collected. A battery of laboratory tests was conducted to exclude metabolic, toxic, and inflammatory causes. A total of 325 Chinese healthy adults matched for gender, age, and residence areas were also recruited as the control group. All the control subjects were examined by neurologists to rule out neurological disorders. All patients’ samples were obtained after a written consent signed by each individual in compliance with the bioethics laws of China, as well as the Declaration of Helsinki. The research was approved by the ethics committee of the first affiliated hospital of Nanchang university.

### Repeat‐primed PCR

The repeat‐primed PCR (RP‐PCR) was initially used to identify the repeat expansion in the *NOTCH2NLC* gene. RP‐PCR was performed as described in our previous study.^4^ In brief, the PCR primer mix contained three primers: 0.3 μmol/L of NOTCH2NLC‐F: 5′‐FAM‐GGCATTTGCGCCTGTGCTTCGGACCGT‐3′, 0.15 μmol/L of M13‐(GGC)4(GGA)2‐R: 5′‐CAGGAAACAGCTATGACCTCCTCCGCCGCCGCCGCC‐3′, and 0.3 μmol/L of M13‐linker‐R: 5′‐CAGGAAACAGCTATGACC‐3′. After incubation at 98°C for 10 min, the cycling conditions were followed by: 16 cycles of 98°C for 30 sec, 66°C for 1 min with reduced 0.5°C per cycle and 68°C for 8 min; then 32 cycles of 98°C for 30 sec, 58°C for 1 min, and 68°C for 8 min; then a final elongation step of 68°C for 10 min. Electrophoresis was performed on a 3500xl Genetic analyzer (Thermo Fisher Scientific, Waltham, MA) and the data were analyzed using GeneMapper software (Thermo Fisher Scientific). A saw‐tooth tail pattern in the electropherogram was considered to be the disease‐associated repeat expansion.

### Nanopore long‐read sequencing

In order to precisely calculate and verify the number of GGC repeat, long‐read sequencing (LRS) was performed on all patients with RP‐PCR‐positive GGC repeat expansion, five patients with RP‐PCR‐negative GGC repeat expansion, and five control subjects through the Oxford Nanopore platform. Large insert‐size libraries were created according to the manufacturer’s protocol (Oxford Nanopore, UK). Mapping‐based methods were used for regular structural variation calling and repeatHMM algorithm was applied for counting GGC motifs in the short tandem repeat region (STR) in the *NOTCH2NLC* gene.

### Pathological examinations of skin biopsy

Open skin biopsies from the distal part of the right leg (10 cm above the external malleous) were performed in two MSA patients (patient 1 and patient 4) with GGC repeat expansions of the *NOTCH2NLC* gene. A part of the specimen was fixed by 4% formalin solution, embedded in paraffin, cut into 4‐mm‐thick sections, and stained with hematoxylin and eosin (HE). The immunohistochemical stains were performed with anti‐p62 antibody (ab56416, Abcam) and alpha‐synuclein (ab27766, Abcam). For electron microscopy, a part of specimens was initially fixed in 2.5% glutaraldehyde, subsequently fixed in 1% osmium tetroxide, and embedded in Epon 812. Ultrathin sections were examined through electron microscope (JEOL‐1230, Japan).

## Results

### GGC expansion in sporadic MSA

**Figure 1:**
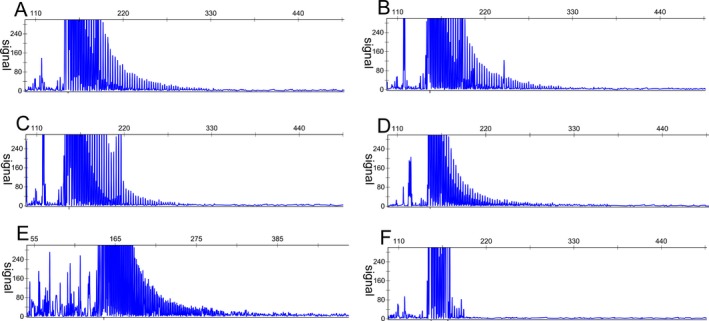
Repeat‐primed PCR was used to identify the repeat expansion in the *NOTCH2NLC* gene. The long saw‐tooth curves indicated that the numbers of GGC in the five patients did exceed a value of at least 100 repeat expansions, A to E stand for patient 1 to patient 5, respectively. The sequencing map showed a single peak wave without saw‐tooth pattern in a healthy control subject (F).

Among 189 patients with Gilman‐criteria MSA, we identified five patients (2.6%) with GGC repeat expansion in the *NOTCH2NLC* gene. The long saw‐tooth curves indicated that the number of GGC in the five patients did exceed a value of at least 100 repeat expansions, although our method could not determine the precise number of GGC repeat expansion (Fig. 1A–E). Among the 325 healthy control subjects, the sequencing map showed a single peak wave without saw‐tooth pattern (Fig. 1F).

**Figure 2:**
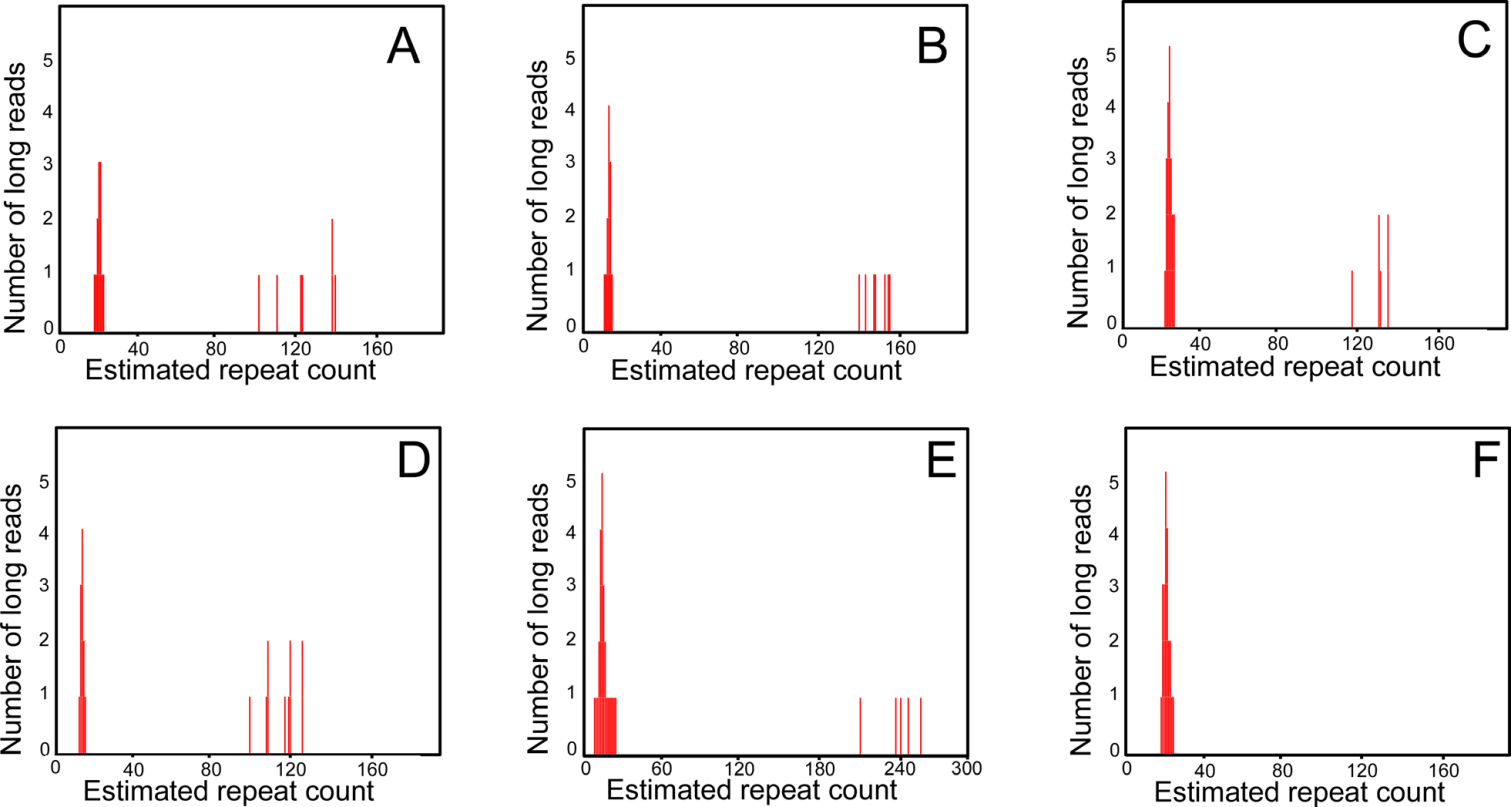
Nanopore long‐read sequencing (LRS) verified the GGC repeat expansion of *NOTCH2NLC*. LRS revealed 12 reads of repeats between 18 and 24, and 7 reads of repeats between 112 and 138 in patient 1(A). LRS showed 14 reads of repeats between 11 and 16, and 7 reads of repeats between 140 and 155 in patient 2 (B). LRS exhibited 21 reads of repeats between 22 and 28, and 6 reads of repeats between 118 and 135 in patient 3 (C). LRS identified 11 reads of repeats between 12 and 16, and 10 reads of repeats between 101 and 126 in patient 4 (D). LRS found 26 reads of repeats between 9 and 23, and 5 reads of repeats between 210 and 266 in patient 5 (E). LRS only showed 21 reads of repeats between 18 and 26 in a control (F).

To confirm the RP‐PCR results and quantify the number of repeat expansion, we performed low‐coverage long‐read genome sequencing. The repeatHMM algorithm was used to count GGC repeats in the STR region of the *NOTCH2NLC* gene. In patient 1, 19 reads of GGC repeats were found in the STR of the *NOTCH2NLC* gene with 12 reads of repeats between 18 and 24, as well as 7 reads of repeats between 112 and 138 (Fig. 2A). In patient 2, repeatHMM identified 21 reads of GGC repeats, including 14 reads of repeats between 11 and 16, and 7 reads of repeats between 140 and 155 (Fig. 2B). In patient 3, 27 reads of GGC repeats were identified, including 21 reads of repeats between 22 and 28, and 6 reads of repeats between 118 and 135 (Fig. 2C). In patient 4, 21 reads of GGC repeats were found, including 11 reads of repeats between 12 and 16, and 10 reads of repeats between 101 and 126 (Fig. 2D). In patient 5, repeatHMM identified 31 reads of GGC repeats, including 26 reads of repeats between 9 and 23, and 5 reads of repeats between 210 and 266 (Fig. 2E). In five patients with RP‐PCR‐negative GGC expansion and five control subjects, repeatHMM indicated GGC repeats ranging between 8 and 29 (Fig. 2F).

### Clinical features of patients with GGC expansion

A total of 189 patients including 101 males and 88 females were recruited in the study. The median age of disease onset was 54 years (interquartile range between 47.5 and 56.5). The median duration of disease at entry was 3 years (interquartile range between 1 and 4.5). Onset of either motor symptoms or dysautonomia was defined as the disease onset. According to the 2008 consensus criteria, 133 (70.4%) patients were diagnosed with probable MSA and 56 (29.6%) patients were diagnosed with possible MSA. Among the 133 patients with probable MSA, 92 (69.2%) patients were classified as MSA‐C and 41 (30.8%) as MSA‐P. Among the 56 patients with possible MSA, 26 (46.4%) patients were classified as MSA‐C and 19 (33.9%) as MSA‐P; 11 (19.7%) could not be categorized into either MSA‐C or MSA‐P.

Five patients with GGC repeat expansion comprised three women and two men (Table 1). The median age of onset was 57 years (interquartile range between 55 and 61) and disease duration at entry was 8 years (interquartile range between 6 and 9). All of them initially presented with prominent urinary dysfunction (four with urinary retention and one with urinary incontinence). According to the 2008 consensus criteria, patient 1 was a probable MSA‐P characterized by orthostatic hypotension, urinary incontinence, poorly levodopa‐responsive parkinsonism, and pyramidal signs; patient 2 was a possible MSA‐C characterized by urinary retention, gait ataxia, tremor, and stridor; patient 3 was a probable MSA‐C characterized by orthostatic hypotension, urinary retention, cerebellar ataxia, and pyramidal signs; patient 4 was a probable MSA‐C characterized by orthostatic hypotension, urinary retention, and cerebellar ataxia; and patient 5 was a probable MSA‐P characterized by orthostatic hypotension, urinary retention, and poorly levodopa‐responsive parkinsonism. A comparison of clinical features between patients with GGC repeat expansion and those without GGC repeat expansion was presented in Table 2. The statistical tests in Table 2 were nonparametric; and the results were exploratory, and were not corrected for multiple comparisons.

**Table 1:** The clinical features of five MSA patients with GGC repeat expansion in the *NOTCH2NLC* gene

Variables	Patient 1	Patient 2	Patient 3	Patient 4	Patient 5
Clinical					
Age(y)/gender	61/F	55/F	57/M	66/F	49/M
Disease duration (y)	9	11	6	8	3
Orthostatic hypotension	+	−	+	+	+
Bladder dysfunction	+	+	+	+	+
Cerebellar ataxia	−	−	+	+	−
Parkinsonism	+	−	−	−	+
Pyramidal signs	+	−	+	−	+
Stridor	−	+	−	−	−
Constipation	+	+	−	−	−
Tremor	+	+	−	+	−
Cognitive impairment	+	+	+	+	−
Muscle weakness	−	−	+	−	−
Visual loss	+	−	−	−	−
Radiological					
Cerebellar atrophy	−	+	+	+	−
Putamen hyperintensity	+	−	−	−	−
Hot cross bun sign	−	−	+	−	−
MCP atrophy	−	−	+	+	−
Cerebellar WML	−	−	+	+	−
Cerebral WML	+	−	+	+	+
Corticomedullary DWI	−	−	−	+	−
GGC repeats range	112–138	140–155	118–135	101–126	210–266
MSA diagnosis	probable	possible	probable	probable	probable
MSA subgroup	MSA‐P	MSA‐C	MSA‐C	MSA‐C	MSA‐P

WML, white matter lesion; DWI, diffused weighted image; MCP, middle cerebellar peduncle; MSA‐C, MSA with predominantly cerebellar ataxia; MSA‐P, MSA with predominantly parkinsonism.

**Table 2:** Comparison of demographic and clinical characteristics between MSA patients with and without GGC repeat expansion.

Variables	Patients with GGC repeat expansion (*n* = 5)	Patients without GGC repeat expansion (*n* = 184)	*P* value*
Gender (male)	2 (40.0%)	99 (53.8%)	0.665
Age (years)	57 (55, 61)	54 (47.5, 56.5)	0.896
Disease duration (years)	8 (6,9)	3 (1, 4.5)	0.045*
Parkinsonism	2 (40.0%)	115 (62.5%)	0.371
Ataxia	3 (60.0%)	157 (85.3%)	0.169
Bladder dysfunction	5 (100.0%)	151 (82.1%)	0.589
Orthostatic hypotension	4 (80.0%4)	139 (75.5%)	1.000
Constipation	2 (40.0%)	88 (47.8%)	1.000
Pyramidal signs	3 (60.0%)	73 (39.7%)	0.393
Cognitive impairments	4 (80.0%)	118 (62.4%)	0.657
Cerebral WML	4 (80.0%)	53 (34.2%)	0.030*
Cerebellar abnormality	3 (60.0%)	139 (75.5%)	0.600
Hot cross bun sign	1 (20.0%)	41 (22.2%)	1.000
MSA‐P	2 (40.0%)	58 (31.5%)	0.653
MSA‐C	3 (60.0%)	115 (62.5%)	0.654
Probable MSA	4 (80.0%)	129 (70.1%)	1.000
Possible MSA	1 (20.0%)	55 (29.9%)	1.000

Variables are presented as *n* (%) in nominal data or median (IQR) in continuous data. For between‐group comparisons, using nonparametric rank sum test or Fisher exact test, as appropriate.

* Denotes a statistically significant difference bettween the two groups, *P* < .05.

**Figure 3:**
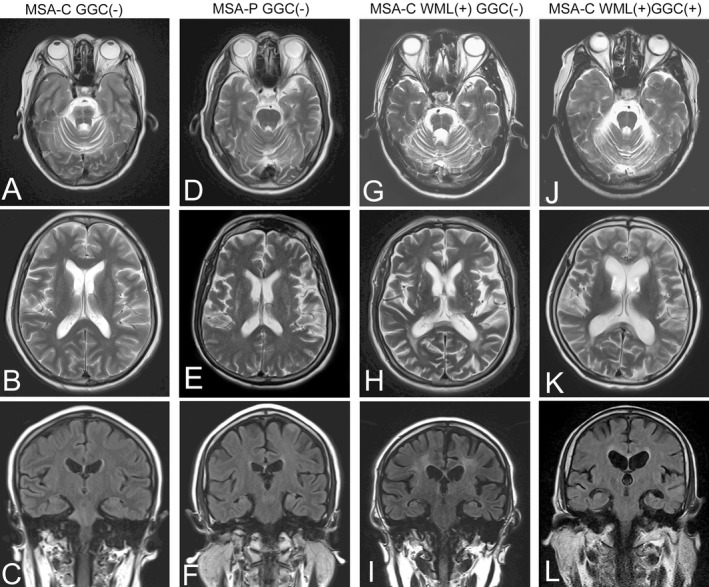
Magnetic resonance imaging representative in this group of MSA patients. Cerebellar atrophy and hot cross bun sign were indicated in a MSA‐C patient without GGC expansion (A–C). Putamen atrophy and lineal T2 high intensity of the lateral margin of the putamen were observed in a MSA‐P patient without GGC expansion (D–F). White matter lesions (WML) suggesting chronic ischemic arteriopathy were notable in a MSA‐C patient without GGC expansion (G–I). White matter lesions and cortical atrophy were found in a MSA‐C patient with GGC expansion (J–L).

Magnetic resonance imaging (MRI) was conducted in all patients, but 91 (48.1%) patients were performed on a 1.5T scanner. Cerebellar atrophy in different degree was identified in 141 patients, and hot cross bun sign was indicated in 42 patients without GGC expansion (Fig. 3A–C). Basal ganglia changes such as putamen atrophy or lineal T2 high intensity of the lateral margin of the putamen were observed in 38 patients without GGC expansion (Fig. 3D–F). In addition, 53 of 184 patients without GGC expansion exhibited periventricular white matter hyperintensities in different degree possibly suggesting chronic ischemic arteriopathy (Fig. 3G–I). In contrast, four of five patients with GGC repeated expansion showed cerebral white matter lesion (Fig. 3J–L).

### Skin pathological changes

**Figure 4:**
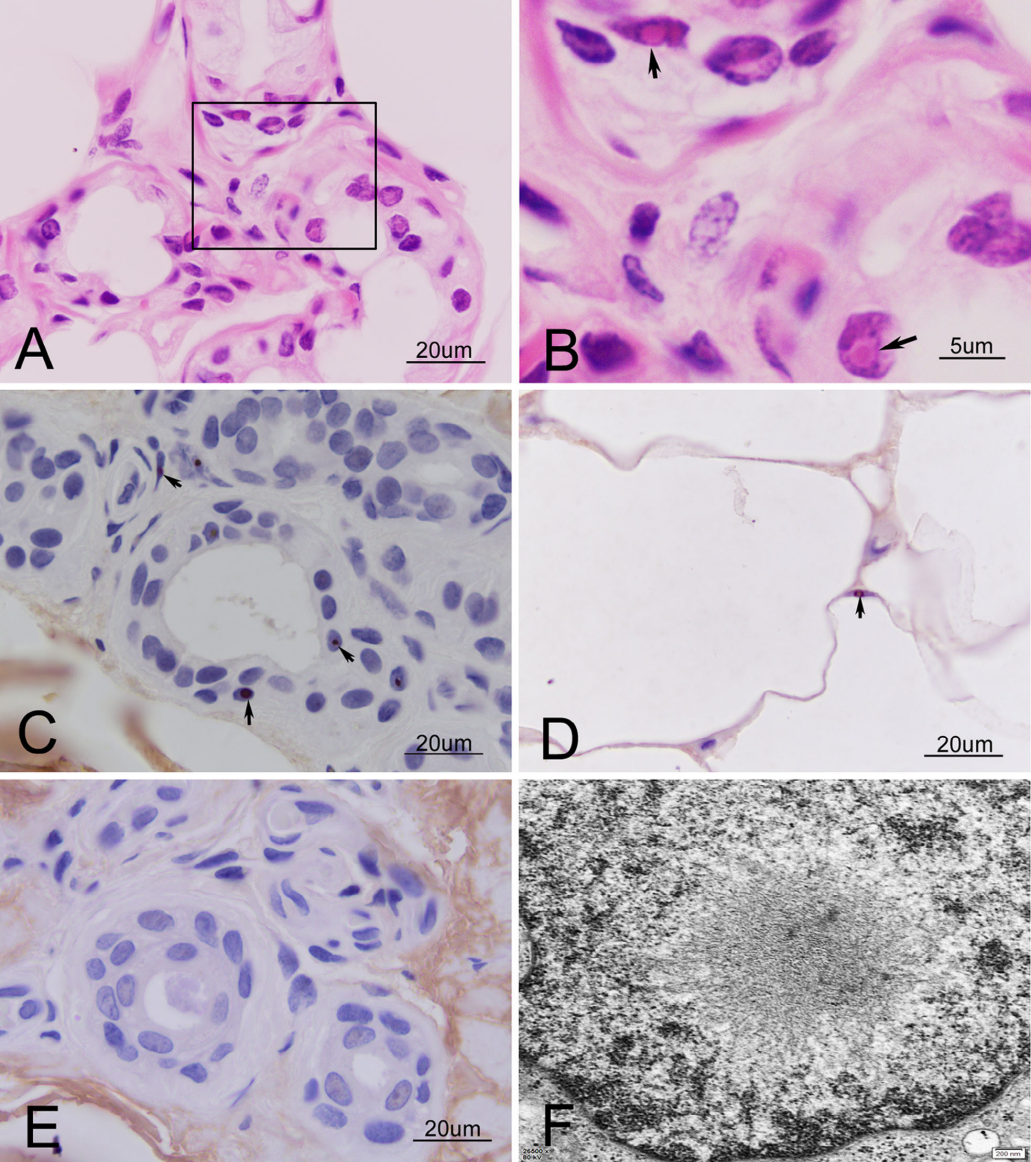
Skin biopsy of two MSA patients with GGC repeat expansion. HE stain revealed typical eosinophilic intranuclear inclusions in the nuclei of fibroblasts and ductal epithelial cells of sweat glands (A). Magnification of the rectangle in A (B, arrow). These eosinophilic inclusions in fibroblasts and ductal epithelial cells of sweat glands (C, arrow) and fat cells (D, arrow) were p62 positive, but alpha‐synuclein negative (E). Electron microscopy revealed a pile of round‐halo filamentous materials in the center of the nucleus (F).

Skin biopsy was performed on two of five MSA patients with GGC repeat expansion. Intriguingly, their skin biopsy samples contained typical eosinophilic intranuclear inclusions in the fibroblasts, fat cells, and ductal epithelial cells of sweat glands (Fig. 4A and B). These eosinophilic inclusions in ductal epithelial cells of sweat glands (Fig. 4C) and fat cells (Fig. 4D) were p62 positive, but alpha‐synuclein negative (Fig. 4E). Electron microscopy revealed a pile of round‐halo filamentous materials in the center of the nucleus (Fig. 4F).

## Discussion

Because of the potential clinical overlap between MSA and NIID,^11^, ^14^ there has been considerable interest in the possibility that the signs and symptoms of some MSA patients may actually be due to GGC repeat expansion of the *NOTCH2NLC* gene. In this study, we used a convenient RP‐PCR to screen the 5′UTR of the *NOTCH2NLC* gene, and found that 2.6% (5/189) of patients with clinically diagnosed MSA had GGC repeat expansions. The results were verified in a part of patients by long‐read sequencing; nevertheless, the incidence would be more convincing, if the repeat number of trinucleotide GGC could be quantified in all subjects recruited in this study.

Familial clustering has been observed in a small number of patients with pathologically proven MSA,^15^, ^16^ although MSA is classically considered as a sporadic disease, evenly a family history of parkinsonism or ataxia is defined as a nonsupporting feature in the current diagnostic criteria.^9^ Neurogeneticists have never given up the hope of identifying potential pathogenic genes for MSA. In 2013,^17^ genetic variants in the *COQ2* gene were associated with familial MSA. The study additionally described several rare variants that were associated with sporadic MSA.^17^ However, the significance of these findings remains unclear because several studies have not confirmed or only partly replicated the findings.^18^ For the same reason, the association between MSA and trinucleotide expansion of *NOTCH2NLC* identified in this study should also be interpreted with cautions, and more studies should be investigated in other patients with clinically diagnosed or pathologically proven MSA.

NIID has been described as a heterogeneous disease because of the variable phenotypes depending on the different patterns of central, peripheral, and autonomic nervous system involvement.^6^, ^19^ Some patients with adult‐onset NIID presented with isolated urinary disturbance at the early stage of disease, and gradually started showing parkinsonism, cerebellar ataxia, dementia, and pyramidal symptoms at the late stage of disease. Therefore, the clinical features in these patients with adult‐onset NIID might mimic the phenotype of MSA.^10^, ^11^ The five MSA patients with GGC repeat expansion in *NOTCH2NLC* had some clinical features that might be useful to differentiate between patients with adult‐onset NIID and MSA.

MSA is a relentlessly progressive neurodegenerative disorder with median survival from symptom onset of 6–10 years.^12^, ^20^ In this study, despite most patients with *NOTCH2NLC* expansion fulfilled Gilman consensus criteria for probable rather than possible MSA, they progressed much more slowly than usual in MSA, and had relatively longer disease duration before diagnosis. Therefore, genetic screening of the *NOTCH2NLC* gene should be recommended for MSA patients with long duration of disease.

Dysautonomia is the most common feature in both MSA and NIID.^6^, ^21^ Patients with MSA usually present with constipation, erectile dysfunction, urinary disturbance, and orthostatic hypotension,^21^ whereas patients with adult‐onset NIID more frequently exhibit bladder dysfunction, miosis, intestinal paralysis, and orthostatic hypotension.^6^ In this study, all five patients showed that bladder dysfunctions were more prominent than other autonomic impairments. In addition, urinary retention was the most frequent urinary feature in these patients; whereas urinary incontinence was documented less commonly. Recently, the long‐term history of neurogenic urinary disturbance prior to other neurological symptoms was emphasized in the diagnostic workflow for adult‐onset NIID.^22^ Therefore, GGC repeat expansion of *NOTCH2NLC* should be reminded in these MSA patients with prominent urinary retention.

Although notable dementia is a nonsupporting feature in the current diagnosis criteria for MSA, cognitive impairment is increasingly recognized as a relevant symptom in MSA patients.^9^, ^21^ Up to 90% of adult‐onset NIID patients are characterized by obviously progressive cognitive impairment.^6^ Our study revealed that patients with repeat expansion in *NOTCH2NLC* presented with prominent cognitive impairment, which might be an important symptom of differential diagnosis between MSA and NIID before performing a genetic test.

Previous studies demonstrated that DWI high signals along the corticomedullary junction and remarkable diffuse T2 high‐intensity signals of the cerebral white matter were characteristic findings in patients with NIID.^6^, ^13^ However, a recent study revealed that only about 37.5% of affected familial individuals presented with the classical NIID radiological findings of linear DWI and severe white matter hyperintensity.^3^ In contrast, MRI studies showing white matter lesions typical of multiple sclerosis other than those commonly seen in this age group are exclusionary red flag in the Gilman criteria for MSA.^9^ Although 34.2% of MSA patients had white matter hyperintensities in our study, the white matter lesions usually distributed anterior and posterior horns of lateral ventricle, which appeared to be nonspecific and commonly observed in older patients ascribed to small vessel disease. In this study, only one patient had mild hyperintense linear lesions in the corticomedullary junction in DWI, four other patients with GGC expansion had white matter hyperintensities, although the white matter lesions were also located at periventricular areas, and might be unrelated to the GGC expansion. Therefore, the NIID patients in the early stage may have been diagnosed as MSA because they did not have typical radiological features of NIID. When patients with MSA showed white matter damage, a differential diagnosis of NIID should be considered.

MSA can be difficult to differentiate from some neurodegenerative disorders such as Parkinson’s disease, spinocerebellar ataxia, and fragile X‐associated tremor ataxia syndrome (FXTAS).^14^, ^23^ Therefore, a definite diagnosis of MSA only can be achieved by pathological autopsy characterized with the loss of neural cells in the central nervous system and by the presence of glial cytoplasmic inclusions in oligodendrocytes throughout the white matter.^20^ As the brains of MSA patients, NIID brains also include multiple ubiquitin‐ and p62‐positive inclusions.^24^ However, the inclusions of MSA should be in oligodendrocytes and contain alpha‐synuclein as a major component, while our study revealed that the inclusions from skin biopsy appeared to be exclusively intranuclear and synuclein negative, rendering a diagnosis of MSA doubtfully. Nevertheless, skin biopsy for alpha‐synuclein appears to have higher sensitivity when acquired in the neck and upper limbs. The lack of alpha‐synuclein in the lower leg sample does completely not exclude superimposed alpha‐synucleinopathy.

Recent studies revealed a broadened clinical spectrum of adult‐onset NIID. GGC repeat expansion was not only identified in typical adult‐ or juvenile‐onset NIID patients^4^ but also observed in Alzheimer disease (AD),^3^ Parkinson disease (PD),^3^ adult leukoencephalopathy,^25^ and essential tremor (ET),^26^ implicating that GGC repeat expansions in the *NOTCH2NLC* gene could partly contribute to the pathological process of multiple neurodegenerative diseases, including MSA, AD, PD, ET, and leukoencephalopathy. Therefore, we also agree to define a term NIID‐related disorders (NIIDRD),^3^ which include NIID and other related neurodegenerative diseases caused by the GGC repeat expansion, although it might be a misdiagnosis in nature.

In summary, adult‐onset NIID should be taken into account for differential diagnosis of MSA. 2.6% of patients with clinically diagnosed MSA had GGC repeat expansions in the 5′UTR of the *NOTCH2NLC* gene. To our knowledge, GGC repeat expansions of the *NOTCH2NLC* gene have not been observed previously in MSA patients. Whether this possible association holds true for a larger number of MSA patients, especially in pathologically proven MSA, remains to be determined.

## Author Contributions

FP, YY, YS, CS, WL, and WH contributed to the acquisition and analysis of data. ZM, CY, and ZK performed the genetic analysis and pathological study. XL and HT contributed to critical revision of the manuscript. HD contributed the study design and drafting the manuscript.

## Conflict of Interest

None declared.

## Ethics Approval

The research was approved by the ethics committee of the first affiliated hospital of Nanchang university (E‐2019‐102).

## Provenance and peer review

Not commissioned; externally peer reviewed.

## Data Availability

All relevant data are described within the paper. Deidentified data can be requested. Data can be requested by all interested researchers, who can be contacted via the corresponding author.
